# Early Intervention in Septic Arthritis of the Hand, Optimizing Patient Outcomes in Hand Infections—A Five-Year Retrospective Study

**DOI:** 10.3390/medicina60060895

**Published:** 2024-05-28

**Authors:** Florin-Vlad Hodea, Andreea Grosu-Bularda, Razvan Nicolae Teodoreanu, Andrei Cretu, Vladut-Alin Ratoiu, Ioan Lascar, Cristian-Sorin Hariga

**Affiliations:** 1 Department 11, Discipline Plastic and Reconstructive Surgery, Bucharest Clinical Emergency Hospital, University of Medicine and Pharmacy Carol Davila, 050474 Bucharest, Romania; florin-vlad.hodea@drd.umfcd.ro (F.-V.H.); razvan.teodoreanu@umfcd.ro (R.N.T.); ioan.lascar@umfcd.ro (I.L.); cristian.hariga@umfcd.ro (C.-S.H.); 2 Clinic of Plastic Surgery, Aesthetic and Reconstructive Microsurgery, Emergency Clinical Hospital Bucharest, 050474 Bucharest, Romania; andrei.cretu@rez.umfcd.ro (A.C.); vladut-alin.ratoiu@rez.umfcd.ro (V.-A.R.)

**Keywords:** septic arthritis, hand infection, joint, infection, upper limb, outcome

## Abstract

*Background and Objectives*: Hand septic arthritis is a potentially debilitating condition that can significantly affect patient functionality and quality of life. Understanding the demographic, clinical, and microbiological characteristics of this condition is crucial for its effective treatment and management. This study aimed to analyze the demographic and clinical profiles of patients with hand septic arthritis, to identify common microbial pathogens, and to evaluate the impact of various factors on clinical course and treatment outcomes. *Material and Methods:* This cross-sectional retrospective study examined patients diagnosed with septic arthritis of the hand, focusing on their demographic data, clinical presentation, causative organisms, treatment methods, and outcomes. Data on age, sex, cause of infection, affected sites, surgical interventions, microbiological findings, and patient outcomes were also collected. *Results:* This study found a higher prevalence of septic arthritis in males and identified bite as the predominant cause. *Staphylococcus aureus* is the most common pathogen. A large number of patients did not exhibit bacterial growth, and bacterial resistance did not significantly affect the outcome. Outcomes were statistically influenced by the timing of medical presentation and the presence of comorbidities. *Conclusions:* Early diagnosis and intervention are critical for effective management of hand septic arthritis. This study underscores the need for a comprehensive approach that considers patient demographic and clinical characteristics to optimize treatment outcomes. Awareness and preventive measures are essential to reduce the incidence and severity of this condition.

## 1. Introduction

Septic arthritis of the hand represents a complex and serious condition that poses significant challenges in terms of diagnosis, treatment, and rehabilitation, affecting a wide range of demographics with various risk factors. Septic arthritis of the hand and wrist affects the adult population and presents a debilitating outcome owing to the severity of the disease. Failure to promptly manage these conditions may lead to impaired joint function or may even necessitate amputation of the finger [1]. It is the second most common type of arthritis, after knee joint afflictions [2,3]. The traumatic event in acute septic arthritis varies and depends on multiple factors, such as the pathogen agent, patient comorbidities, and the timeline from insemination to presentation, therefore imposing different surgical treatment methods ranging from simple incision, debridement, and evacuation up to joint resection with arthrodesis and, more severely, amputation. In selected cases, secondary arthroplasty is warranted to restore function [4,5,6].

Functional recovery of joints after infection and surgical intervention varies based on the aforementioned factors and therapeutic results. Regardless of joint-sparing results, joint stiffness poses a problem and will require rehabilitation, and patients with poor prognosis may need a rehabilitation protocol to achieve the most favorable outcome [7,8].

This disease significantly affects the professional lives of patients in a highly active functional age group, which may present with a permanently debilitating deficit, resulting in a significant loss of function [9,10].

This cross-sectional retrospective study aimed to comprehensively analyze the epidemiological and clinical aspects of septic arthritis in the hand and wrist joints, considering patient demographics, presence of comorbidities, presentation date from the traumatic agent, anatomical involvement, and characteristics of bacterial involvement.

The relationship between patient comorbidities, the timing of medical presentation post-symptom onset, and the type of bacterial growth was explored. Our goal was to determine whether these factors correlate with the severity of outcomes, which, in our study, ranged from maintaining joint integrity to more severe interventions, such as joint resection with arthrodesis or even amputation. Our study aimed to provide valuable insights into the management of septic arthritis of the hand and wrist, potentially guiding future therapeutic strategies to mitigate severe outcomes.

## 2. Materials and Methods

A 5-year retrospective cross-sectional study was performed on patients admitted to the Clinic of Plastic and Reconstructive Surgery at the Clinical Emergency Hospital Bucharest, Romania, a tertiary health center, between 2018 and 2022. The inclusion criteria were adult patients with acute septic arthritis symptoms of the hand and wrist who were admitted to the emergency department. Exclusion criteria were relapse of chronic inflammatory diseases of the joints, insufficient data pertaining to study variables, and involvement of anatomical areas other than the hands and wrists.

Written consent was obtained from all the patients during admission. Confidentiality and data protection is ensured, respecting hospital protocols for ethical guidelines, and receiving ethics approval (number 2579) on 26 March 2024. Data collection involved accessing both the physical and digital archives of the hospital records. To reduce bias, data were reviewed and cross-verified by several individuals.

A total of 118 patients were included in this study. However, the patient group underwent several reductions in numbers for various reasons. Specifically, two of these patients left before completing the treatment. Additionally, nine patients were identified as having chronic recurring arthritis, a condition that fell outside the scope of the study. Furthermore, 33 patients were excluded because their medical records or data were incomplete or the anatomical site did not match the purpose of the study. Consequently, these exclusions resulted in a refined and more specific patient group for analysis.

The treatment algorithm involved thorough history, physical examination, and imaging (radiography). As per the hospital protocol, all patients with septic arthritis were admitted and received dual treatment, with the initiation of empiric intravenous antibiotics and surgical incision, irrigation, and debridement. Antibiotic administration was adjusted based on the antibiogram.

Recorded and investigated data included demographic characteristics, etiologic agents, days from traumatic event to presentation, comorbidities, culture results, antibiotic resistance, joint location of infection, and type of surgical decision. Analysis was performed using Microsoft Excel v.2021 and IBM SPSS statistics v.26, using descriptive analysis and Chi-Square and Fisher’s exact tests for statistical analysis. The findings were then compared with those of existing research in the Web of Science, PubMed, and Google Scholar databases.

## 3. Results

**Figure 1:**
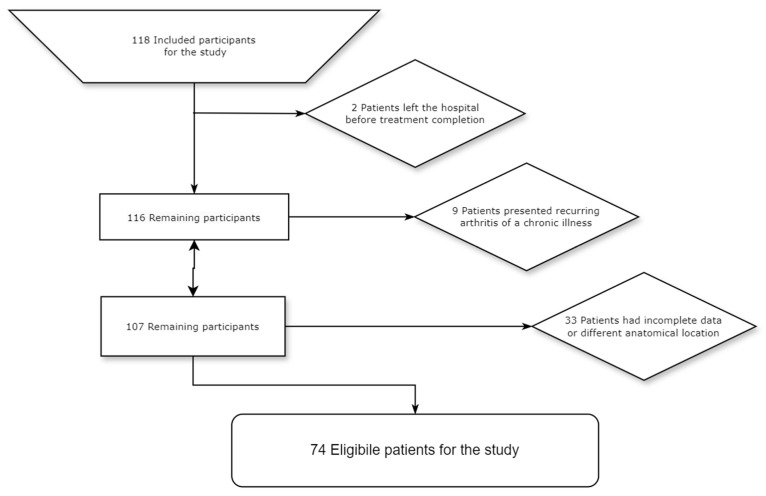
Flowchart of patient eligibility.

Potential eligible participants for this study were patients admitted to the Clinic of Plastic and Reconstructive Surgery in the Clinical Emergency Hospital Bucharest, Romania, within a 5-year timeframe between 2018 and 2022. A total of 118 patients were admitted according to the hospital protocol, each undergoing one or multiple interventions. A total of 118 patients were included in this study. Two patients were discharged before the completion of treatment. Nine patients presented with chronic recurring or inflammatory arthritis, and 33 patients either did not present the anatomical site that matched the purpose of the study for hand and wrist evaluation or had incomplete data. Consequently, 74 patients were eligible for this study, as shown in the flowchart in Figure 1.

In this study demographics analysis revealed that most patients were male with an average age of around 51.4 years, exhibiting a standard deviation of 17.7 years in age distribution. Their ages ranged from 19 to 86 years. Specifically, 25% of the participants were under 38 years of age, the median age was 51 years, and 75% were younger than 64 years. The data revealed notable differences in injury distribution, with younger patients and males predominating. The metacarpal–phalangeal (MP) joint was the most commonly affected joint, followed by the proximal interphalangeal (PIP) joint, whereas the radiocarpal joint (RCJ) and thumb interphalangeal (IP) joints were less commonly involved, as detailed in Table 1. The thumb had seven cases of MP involvement and nine cases of PIP involvement. The index finger had 9 cases of MP, 10 cases of PIP, and 2 cases of DIP involvement. The third finger had 10 cases of MP, 5 cases of PIP, and 5 cases of DIP involvement. The fourth finger had three cases of MP, three cases of PIP, and one case of DIP involvement. Lastly, the fifth finger had one case of MP, six cases of PIP, and one case of DIP involvement.

The analysis of etiologies, as seen in Table 2, leading to septic arthritis of the hand revealed that bites were the most frequent cause, accounting for nearly half of the cases. Dog bites were the most common, closely followed by cat and human bites, while pig bites were rare. Despite being the most common bite injury, not all bite injuries result in severe outcomes. A significant number of these cases had favorable outcomes, particularly human bites, which showed a higher rate of positive recovery than others.

Cuts from handheld electric tools, glass, knives, and other sharp objects were the second most common cause of injury. These types of injuries vary in severity, with a notable proportion leading to favorable outcomes. Electric handheld tools are frequently implicated in severe injuries within this category.

Stings from objects, plants, and insects constituted a significant proportion of the cases. While most stings led to favorable outcomes, there were instances in which the injuries were severe. A small percentage of injuries were categorized as "unknown" because of the inability to determine the exact cause. These cases included a mix of outcomes, some of which resulted in severe consequences.

Additionally, there were specific instances of foreign object retention in the joints, which resulted in favorable outcomes. However, conditions such as arthritic changes secondary to felons, crush injuries, falls, self-injection of drugs, and open-joint dislocations generally lead to severe outcomes.

The presence of comorbidities significantly influenced the patient outcomes. Generally, patients without any comorbidities had better recovery rates than those with comorbidities. Among the patients with comorbidities, those with a single condition fared better than those with multiple comorbidities.

The most prevalent comorbidities, as noted in Table 3, were arterial hypertension, diabetes mellitus, and chronic kidney disease (CKD). Patients with arterial hypertension made up a substantial proportion, with a notable percentage of patients experiencing severe outcomes. Similarly, diabetes mellitus was prevalent among patients and strongly associated with severe outcomes, highlighting the critical impact of this condition on recovery.

Other comorbidities include viral hepatitis, alcohol abuse disorders, autoimmune diseases, and neoplasms. While viral hepatitis and alcohol abuse disorders were less common, they still contributed to severe outcomes in some cases. Chronic kidney disease and autoimmune diseases were particularly concerning, as all cases within these categories resulted in severe outcomes, underscoring their severity.

Additionally, heart disease and HIV infection were identified in the patients. Heart disease cases had favorable outcomes, whereas HIV cases were split between favorable and severe outcomes. The presence of neoplasms also has a mixed impact, with some patients recovering well and others experiencing severe complications.

Of the 74 patients, a significant portion did not show any growth in microbiological cultures (Table 4). Among those who did, the outcomes were nearly equally distributed between the favorable and severe groups. Bacterial infections were predominantly monomicrobial, with *Staphylococcus aureus* being the most common pathogen identified. Other frequently isolated bacteria include *Enterococcus* spp., coagulase-negative Staphylococcus, and *Escherichia coli*.

Polymicrobial infections, although less frequent, were associated with a higher incidence of severe outcomes compared to monomicrobial infections. Antibiotic resistance is a notable issue, with some bacterial strains identified as multidrug resistant (MDR). *Staphylococcus aureus*, including methicillin-resistant *Staphylococcus aureus* (MRSA), is a common MDR pathogen. Patients with MDR bacteria have mixed outcomes. In contrast, patients with non-resistant bacterial infections tended to have more favorable outcomes.

Overall, while the presence of bacterial infection and antibiotic resistance did not significantly alter the overall distribution of severe versus favorable outcomes, specific factors, such as the type of infection and resistance patterns, played a critical role in patient prognosis. A detailed breakdown of the isolated pathogens reveals the diversity of the bacterial species involved.

The most common surgical intervention was the evacuation of septic collections, thorough irrigation, and debridement, which resulted in joint preservation and favorable outcomes in the majority of cases. In severe cases with significant joint damage, joint resection followed by arthrodesis was performed, and segmental amputation was necessary in some cases.

The timing of presentation after a traumatic event was critical. Early presentation, defined as within seven days, was associated with more favorable outcomes, whereas late presentation, defined as after seven days, often led to severe outcomes (Table 5). To comprehensively assess the factors influencing patient outcomes, we recorded and analyzed variables such as population age, time of presentation to the hospital, presence of comorbidities, culture growth, differentiation between monomicrobial and polymicrobial cultures, and prevalence of antibiotic-resistant bacteria. Our objective was to determine whether these factors had a statistically significant effect on patient outcomes. The data presented in Table 5 underscores the substantial effect of comorbidities and the timing of hospital presentation on patient outcomes. In contrast, other factors, including age, bacterial growth, polymicrobial infections, and antibiotic resistance, did not exhibit a statistically significantly affect the outcomes of this study.

## 4. Discussion

The incidence of septic arthritis of the hand is 2–12 cases per 100,000 individuals per year, second only to knee septic arthritis [1,2]. It represents a medical condition that has a high rate of joint resection followed by arthrodesis or may even require segment amputation in 50–75% of cases [11]. This study analyzed patients with septic arthritis of the hand and wrist over five years, focusing on demographics, injury causes, comorbidities, and microbiological findings. Comorbidities significantly impacted outcomes, with arterial hypertension and diabetes mellitus being the most common comorbidities. Patients with multiple comorbidities have a higher risk of severe outcomes. Microbiological analysis showed that most infections were monomicrobial, with *Staphylococcus aureus* as the predominant pathogen. Polymicrobial infections and antibiotic-resistant bacteria are associated with severe outcomes. Early hospital presentation is crucial for achieving favorable outcomes.

The demographic analysis of patients with acute septic arthritis of the hand and wrist revealed a notable impact on the working-age population, with a significant proportion of patients being labor-active individuals under 64 years of age. This finding underscores the socioeconomic burden of the condition, emphasizing the need for prompt and effective treatment to facilitate a quick return to work and minimize productivity loss. Preventive measures and workplace safety protocols are essential for reducing the incidence of such injuries [12,13].

Old age (>64 years) has been reported as an individual factor predicting more severe outcomes, including an increased risk of mortality. However, in this study, the analysis of age as a risk factor for outcome severity did not yield statistically significant results, with a *p*-value of 0.094498 (*p*-value > 0.05). It has been reported in current data as a risk factor and even a mortality predictor in cases of septic arthritis, leading to poor outcomes in geriatric patients older than 65 years and even more impactful outcomes in patients older than 80 years. Although the data suggest that older age might be associated with severe outcomes, the lack of statistical significance indicates that, within this sample, age alone may not be a definitive predictor of severity in septic arthritis cases. This underscores the need for further research to explore the interplay between age and other factors that influence patient outcomes [14,15].

Comparing the two sexes, we found that the prevalence of septic arthritis in the hand and wrist was higher in males than in females, with a male-to-female ratio of almost 2:1. This finding is consistent with that reported in the literature. For instance, Rotunno et al. (2019) observed a similar male predominance in their dual-center study of septic joint arthritis of the hand, suggesting that men are more frequently affected by this condition. Additionally, Ferrand et al. (2016) reported that the incidence of septic arthritis was higher in males in their three-year hospital-based study, further supporting our findings. Several factors may have contributed to this gender disparity. Men are more likely to engage in activities that increase the risk of hand injuries, such as certain types of manual labor, sports, and hobbies involving tools or machinery [9,16].

A significant proportion of patients with septic arthritis of the hand are active individuals, for whom optimal hand functionality is crucial. This condition poses a considerable challenge, as it often leads to highly debilitating outcomes. Septic arthritis in the hand disrupts not only the physical integrity and functionality of the hand, but also significantly affects the quality of life of patients. This necessitates prompt and effective medical interventions to prevent long-term damage and restore hand functionality [11,17].

The initiation of a prompt and efficient dual therapy regimen, encompassing both intravenous antibiotics and comprehensive debridement of infected tissues, along with antiseptic irrigation, constitutes the cornerstone of treatment for achieving the most favorable outcomes. Initially, intravenous broad-spectrum antibiotics were used until culture results allowed for targeted therapy, enhanced treatment efficacy, and reduced resistance risks.

Comprehensive debridement involves meticulous removal of necrotic and infected tissue from the affected joint, significantly reducing bacterial load. Combined with antiseptic irrigation, which flushes the joint with antiseptic solutions, residual bacteria and debris are eliminated [18,19].

Adequate care for patients with septic arthritis of the hand is essential for their reintegration into social and labor environments, thereby reducing the economic burden associated with this condition. When patients receive timely and appropriate medical interventions, they are more likely to regain full use of their hands, which is critical for performing daily tasks and maintaining employment. Septic arthritis can have debilitating effects on a patient’s ability to work and participate in social activities. Without proper treatment, patients may suffer from chronic pain, reduced mobility, and long-term disability, leading to prolonged absence from work and increased healthcare costs [20,21].

Injury sites among the patients in this study revealed a predisposition of the radial side of the hand to injury, with the second finger accounting for 21 cases and the third finger for 20 cases, together accounting for 55% of cases. This confirms a site predilection consistent with the findings in the literature.

This radial side predilection has significant implications for hand function, particularly in activities requiring radial-sided grips, such as key, tip-precision, and tripodal grips. The second and third fingers are integral to these types of grips, which are essential for tasks that require fine motor skills such as writing, typing, and manipulating small objects. Injury to these fingers can severely impair an individual’s ability to perform these tasks, leading to a substantial impact on their daily living and work activities [2,22,23].

The thumb was the next most frequently involved site after the second and third fingers, whereas the ulnar side of the hand, including the fourth and fifth fingers, was less frequently affected. The metacarpophalangeal (MCP) and proximal interphalangeal (PIP) joints were the most commonly involved joints, likely because of their high exposure and vulnerability to trauma, which serve as entry points for infection [24].

The data also show instances of septic arthritis in the wrist joint, which, while rarer, signifies a more complex clinical scenario, given the wrist’s intricate structure and the potential for significant impairment in function. Septic arthritis of the wrist is rare, with risk factors including advanced age, rheumatoid arthritis, immunodeficiency, and diabetes mellitus. Effective treatment requires a combination of synovectomy, debridement, immobilization, and antibiotics. It is necessary to emphasize that early surgical intervention through arthrotomy, ideally within hours of diagnosis, significantly improves patient outcomes [25,26,27].

Analysis of etiologies revealed a diverse range of causes, with bites being notably predominant and the most frequent traumatic agent.

These types of injuries frequently determine septic arthritis of the hand because of the nature of the pathogens present in the oral flora of animals and humans and the mechanics of the bite, which often results in penetrating injuries that introduce polymicrobial pathogens directly into the joint space [5,28,29,30].

Stings and cuts have the potential to inseminate affected joints directly through the introduction of skin flora, such as *Staphylococcus aureus* and group A β-hemolytic streptococci, within the joint, or to inseminate directly through contamination [5,31,32].

Felons or infections of the fingertip’s pad should not be overlooked because of their capacity to lead to severe complications. These complications include tenosynovitis, osteomyelitis, and septic arthritis. Such severe complications of joint involvement were observed in the three patients included in this study, highlighting the critical nature of timely and effective intervention. The progression from a seemingly localized infection to a more debilitating condition underscores the importance of recognizing and effectively treating felons [33,34].

Notably, in five cases, individuals were unable to identify a specific traumatic event or injury preceding the onset of symptoms, attributing the infection to an unknown source. These patients did not present with any other joint involvement or systematic clinical findings. This observation aligns to some extent with the existing literature, which suggests that up to 10% of septic arthritis cases with unknown causes could stem from hematogenous spread [35,36].

The most frequent surgical intervention for septic arthritis is collection evacuation and debridement, which often result in successful joint salvage and favorable outcomes in most patients. Arthrodesis was performed in cases where the joints were severely damaged, whereas amputation was necessary in situations with extensive joint, bone, and soft tissue damage, where salvage was not feasible. This range of surgical decisions highlights the aggressive and varied nature of managing septic arthritis, emphasizing the severity of the condition and the importance of tailoring treatment strategies to each patient’s specific clinical scenario [37,38].

A significant proportion of patients (33.8%) did not exhibit any bacterial growth in infected synovial fluid. Factors contributing to this lack of growth may include practice-related etiologies such as poor sampling techniques for contaminated joints and poor plating techniques. However, it has been reported that the dominant etiology may be primary-care empirical antibiotic treatment or self-prescribed treatment prior to presentation to our unit. This phenomenon is largely attributed to the prevalent use of antibiotics in Romania, with reports indicating that 3% of the population consume antibiotics daily. Such widespread antibiotic use can preemptively eliminate bacterial evidence and complicate diagnosis [39,40,41].

Based on the data provided, the presence or absence of pathogens did not significantly affect the outcome of patients with septic arthritis. Among the cases in which specific bacteria were identified, the majority were monomicrobial, with a smaller proportion being polymicrobial. The most frequently detected bacterium was *Staphylococcus aureus*, which is consistent with existing literature on septic arthritis. Other common bacteria include Streptococcus and Enterococcus species, followed by coagulase-negative Staphylococcus, *Escherichia coli*, Proteus, and *Klebsiella pneumoniae*. The less frequently identified bacteria were Acinetobacter, *Pasteurella canis*, Citrobacter, Moraxella, and Providencia. These findings highlight *Staphylococcus aureus* as the predominant pathogen, while the presence of Enterococcus and Streptococcus species as significant pathogens contrasts with the literature, which typically notes Pasteurella species as more common secondary pathogens [42,43,44,45].

Among the cases in which specific bacteria were identified, most were monomicrobial (73.5%). A small number of cases involve multiple bacterial species. Comparing monomicrobial to polymicrobial contamination using statistical analysis, the OR (odds ratio) was 3.03, suggesting that patients with polymicrobial contamination are about three times more likely to have a severe outcome compared to those with monomicrobial contamination. However, there is insufficient evidence to conclude that there is a statistically significant association between the type of bacterial contamination and the severity outcome. The literature reports 3.4–11% of cases of polymicrobial septic arthritis, while the current study describes a higher percentage (17.5%) of all cases of septic arthritis [5,46,47,48].

Antibiotic-resistant bacteria were detected in the patients in this study. Most bacterial strains were susceptible to antibiotics, but a subset was multidrug-resistant, including strains of *Staphylococcus aureus*, coagulase-negative Staphylococcus, *Escherichia coli*, *Klebsiella pneumoniae*, Streptococcus beta-hemolytic (A group), Proteus, and Citrobacter. No extensive or pan-drug-resistant bacteria were detected. Among patients with multidrug-resistant bacteria, the outcomes were evenly split between severe and favorable. Similarly, patients with non-resistant bacteria had a mix of severe and favorable outcomes. Statistical analysis revealed no significant differences in patient outcomes based on antibiotic resistance. However, half of the MDR patients had severe outcomes. Antibiotic resistance complicates the treatment process, leading to delays in completing therapy and necessitating the use of more targeted antibiotic regimens guided by culture and sensitivity results. This resistance presents a significant challenge in clinical practice, complicating diagnosis and development of effective treatment strategies [49,50]. ESKAPE pathogens, defined as a group of pathogens consisting of *Enterococcus faecium*, *Staphylococcus aureus*, *Klebsiella pneumoniae*, *Acinetobacter baumannii*, *Pseudomonas aeruginosa*, and *Enterobacter* spp., which reportedly possess the capacity to develop antibiotic resistance and determine complications, were found among the patients in this study but did not influence the severity of the outcome, compared to cases described in the literature [51,52].

The presence of comorbidities significantly influenced the patient outcomes in this study. Patients without any comorbid conditions generally experience better recovery rates than those with existing health issues. Common comorbidities included arterial hypertension, diabetes mellitus, and chronic kidney disease, which is similar to the literature. These conditions compromise the patients’ immune responses, making them more susceptible to severe infections and complicating the management of septic arthritis. Patients with multiple comorbidities face an even higher risk of severe outcomes, highlighting the compounded impact of having several health issues simultaneously. Comorbidities were not only described as a risk factor for the severity of outcomes in the literature but also as a predictor of mortality, as well as a higher risk of hospitalization and longer stay [12,53,54].

The timing of presentation significantly affected the patient outcomes in this study. Patients who sought medical attention early, within seven days of symptom onset, generally experienced more favorable outcomes. Early intervention allowed for prompt initiation of treatment, including antibiotic therapy and surgical intervention, which helped control the infection and prevent extensive joint damage. By contrast, patients who delay seeking medical care often experience more severe complications. Late presentation typically results in more advanced infection, greater tissue damage, and a higher likelihood of requiring more aggressive and extensive surgical procedures such as arthrodesis or amputation. These findings highlight the critical importance of the early recognition and treatment of septic arthritis to optimize patient outcomes and minimize the risk of long-term disability. Educating patients and healthcare providers about the signs and symptoms of septic arthritis can facilitate quicker diagnosis and treatment, ultimately improving the prognosis [55].

While this study considered only the initial management of septic arthritis of the hand and wrist, further interventions are needed. Therefore, secondary amputation or joint resection may be warranted in cases of relapse or improper rehabilitation. The recovery and maintenance of hand function post-infection is critical. An effective program tailored to individual patient needs must be implemented for rapid social and professional reintegration to achieve the best possible functional outcome [11,56,57].

This study has several limitations that should be acknowledged. First, the retrospective study design inherently introduces selection bias as it relies on existing records and data that may not comprehensively represent all cases. The study was conducted within a single healthcare unit, limiting the generalizability of the findings to other settings or populations. Additionally, we did not compile a database for tracking complications, which could have provided valuable insights into the long-term impact and potential sequelae of septic arthritis. The absence of long-term follow-up data further limits our understanding of the patient outcomes beyond the immediate treatment period. Furthermore, we did not collect data on antibiotic use prior to hospital presentation for patients who did not have culture results, which could have influenced the observed bacterial profiles and resistance patterns. These limitations highlight the need for future prospective studies with broader settings, comprehensive complication tracking, and long-term follow-up to provide a more complete picture of the management and outcomes of septic arthritis. Lastly, we did not collect data on patients who presented later and had self-prescribed antibiotics or received them through primary care. The lack of these data may impact the interpretation of treatment outcomes and prognosis, as prior antibiotic use could alter the clinical presentation and response to subsequent treatments.

## 5. Conclusions

There is notable variation in patient age, with a considerable number of cases occurring in the labor-active population, emphasizing the potential economic implications of work-related disabilities. The prevalence of septic arthritis was higher in males than in females, and injuries, such as bites, were identified as common causes, particularly affecting the radial side of the hand.

Surgical management plays a pivotal role in treating septic arthritis, with outcomes heavily influenced by the extent of the initial joint damage and timely initiation of treatment. Concerning the diversity of bacterial etiology, this study found that *Staphylococcus aureus* was most prevalent. Furthermore, a significant proportion of patients showed no bacterial growth in cultures. In addition, we found that bacterial contamination did not affect the outcome of patients with septic arthritis. However, other factors were detrimental, such as the presence of comorbidities and delay in presentation.

Early diagnosis, the presence of comorbidities, and the timing of medical intervention may be the most important criteria for successful patient recovery. This study suggests that a delay in treatment can exacerbate this condition, leading to severe long-term disability. Therefore, a comprehensive, patient-centered approach that considers individual patient histories and specific clinical features is imperative for the effective management of septic arthritis. These findings have substantial implications in clinical practice, emphasizing the need for prompt and accurate diagnosis and tailored treatment strategies to improve patient outcomes.

## Figures and Tables

**Table 1:** Demographics and anatomical site joint injury.

	Total (*n* = 74)	Outcome—Favorable (*n* = 49)	Outcome—Severe (*n* = 25)
Sex, *n* (%)			
Males, *n* (%)	47 (63.5%)	29 (59.2%)	18 (72%)
Females, *n* (%)	27 (36.5%)	20 (40.8%)	7 (28%)
Anatomical site, *n* (%)			
Wrist, *n* (%)	2 (2.7%)	1 (2%)	1 (4%)
Thumb, *n* (%)	16 (21.6%)	9 (18.4%)	7 (28%)
Second finger, *n* (%)	21 (28.4%)	15 (30.6%)	6 (24%)
Third finger, *n* (%)	20 (27%)	12 (24.5%)	8 (32%)
Fourth finger, *n* (%)	7 (9.5%)	5 (10.2%)	2 (8%)
Fifth finger, *n* (%)	8 (10.8%)	7 (14.3%)	1 (4%)
Affected joint, *n* (%)			
RCJ, *n* (%)	2 (2.7%)	1 (2%)	1 (4%)
MPJ, *n* (%)	30 (40.5%)	26 (53%)	4 (16%)
PIPJ, *n* (%)	24 (32.4%)	17 (34.8%)	7 (28%)
DIPJ, *n* (%)	9 (12.2%)	2 (4.1%)	7 (28%)
Thumb IPJ, *n* (%)	9 (12.2%)	3 (6.1%)	6 (24%)

**Table 2:** Etiology of traumatic agents.

Etiology, *n* (%)	Total (*n* = 74)	Outcome—Favorable (*n* = 49)	Outcome—Severe (*n* = 25)
Bites, *n* (%)	33 (44.6%)	24 (49%)	9 (36%)
Bite (Dog), *n* (%)	12 (16.2%)	7 (14.3%)	5 (20%)
Bite (Cat), *n* (%)	10 (13.5%)	8 (16.3%)	2 (8%)
Bite (Human), *n* (%)	10 (13.5%)	9 (18.4%)	1 (4%)
Bite (Pig), *n* (%)	1 (1.4%)	0	1 (4%)
Cuts, n (%)	13 (17.6%)	10 (20.4%)	3 (12%)
Cut (Electric handheld), *n* (%)	4 (5.4%)	2 (4.1%)	2 (8%)
Cut (Unknown), *n* (%)	4 (5.4%)	3 (6.1%)	1 (4%)
Cut (Knife), *n* (%)	2 (2.7%)	2 (4.1%)	0
Cut (Glass), *n* (%)	2 (2.7%)	2 (4.1%)	0
Cut (Axe), *n* (%)	1 (1.4%)	1 (2%)	0
Stings, *n* (%)	11 (14.9%)	9 (18.4%)	2 (8%)
Unknown, *n* (%)	5 (6.75%)	3 (6.1%)	2 (4.1%)
Foreign body retention, *n* (%)	3 (4.1%)	3 (6.1%)	0
Felons, *n* (%)	3 (4.1%)	0	3 (12%)
Crush injury, *n* (%)	2 (2.7%)	0	2 (8%)
Fall injury, *n* (%)	2 (2.7%)	0	2 (8%)
Self-injection, *n* (%)	1 (1.4%)	0	1 (4%)
Open joint dislocation, *n* (%)	1 (1.4%)	0	1 (4%)

**Table 3:** Patient comorbidities.

	Total (*n* = 74)	Outcome–Favorable (*n* = 49)	Outcome—Severe (*n* = 25)
Patients with comorbidities, *n* (%)			
Comorbidities absent, *n* (%)	43 (58.1%)	35 (71.4%)	8 (32%)
Single comorbidity, *n* (%)	18 (24.3%)	5 (10.2%)	13 (52%)
Multiple comorbidities, *n* (%)	13 (17.6%)	9 (18.4%)	4 (16%)
List of comorbidities, *n* (%)			
Arterial hypertension, *n* (%)	17 (23%)	8 (16.3%)	9 (36%)
Diabetes mellitus, *n* (%)	11 (14.8%)	3 (6.1%)	8 (32%)
Viral hepatitis, *n* (%)	4 (5.4%)	3 (6.1%)	1 (4%)
Alcohol abuse disorder, *n* (%)	3 (4%)	1 (2%)	2 (8%)
CKD, *n* (%)	3 (4%)	0	3 (12%)
Autoimmune disease, *n* (%)	3 (4%)	0	3 (12%)
Neoplasm, *n* (%)	2 (2.7%)	1 (2%)	1 (4%)
Heart disease, *n* (%)	2 (2.7%)	2 (4%)	0
HIV, *n* (%)	2 (2.7%)	1 (2%)	1 (4%)

**Table 4:** Microorganism description.

	Total (*n* = 74)	Outcome—Favorable (*n* = 49)	Outcome—Severe (*n* = 25)
Isolated pathogens, *n* (%)	Total (*n* = 59)	Susceptible to antibiotics (*n* = 49)	Multi drug resistant (*n* = 10)
*Staphylococcus aureus*, *n* (%)	15 (25.4%)	12 (24.5%)	3 (15.8%)
Methicillin resistant *Staphylococcus aureus*, *n* (%)	4 (6.8%)	2 (4.1%)	2 (10.5%)
Non-methicillin resistant *Staphylococcus aureus*, *n* (%)	11 (18.6%)	10 (20.4%)	1 (5.3%)
*Enterococcus* spp., *n* (%)	9 (15.3%)	9 (18.4%)	0
Coagulase negative staphylococcus, *n* (%)	6 (10.2%)	4 (8.2%)	2 (10.5%)
*Escherichia coli*, *n* (%)	5 (8.5%)	4 (8.2%)	1 (5.3%)
*Proteus* spp., *n* (%)	4 (6.8%)	3 (6.1%)	1 (5.3%)
*Klebsiella pneumoniae*, *n* (%)	3 (5.1%)	2 (4.1%)	1 (5.3%)
Streptococcus beta hemolytic, A group, *n* (%)	2 (3.4%)	1 (2.0%)	1 (5.3%)
Streptococcus beta hemolytic, F group, *n* (%)	2 (3.4%)	2 (4.1%)	0
*Streptococcus viridans*, *n* (%)	2 (3.4%)	2 (4.1%)	0
Streptococcus group D, *n* (%)	2 (3.4%)	2 (4.1%)	0
*Moraxella* spp., *n* (%)	1 (1.7%)	1 (2.0%)	0
*Acinetobacter Iwoffi*, *n* (%)	1 (1.7%)	1 (2.0%)	0
*Acinetobacter baumannii*, *n* (%)	1 (1.7%)	1 (2.0%)	0
*Citrobacter* spp., *n* (%)	1 (1.7%)	0	1 (5.3%)
*Providencia* spp., *n* (%)	1 (1.7%)	1 (2.0%)	0
Streptococcus beta hemolytic, C group, *n* (%)	1 (1.7%)	1 (2.0%)	0
Streptococcus beta hemolytic, D group, *n* (%)	1 (1.7%)	1 (2.0%)	0
*Pasteurella canis*, *n* (%)	1 (1.7%)	1 (2.0%)	0
Streptococcus, group B, *n* (%)	1 (1.7%)	1 (2.0%)	0

**Table 5:** Factors compared with outcome.

	Total (*n* = 74)	Outcome—Favorable (*n* = 49)	Outcome—Severe (*n* = 25)	
Age, *n* (%)				
Age ≤ 64, *n* (%)	56 (75.7%)	40 (81.6%)	16 (64%)	
Age > 64, *n* (%)	18 (24.3%)	9 (18.4%)	9 (36%)	*p*-value = 0.094498 (*p*-value > 0.05)
Comorbidities, *n* (%)				
Comorbidities absent, *n* (%)	43 (58.1%)	35 (71.4%)	8 (32%)	
Comorbidities present, *n* (%)	31 (41.9%)	14 (28.6%)	17 (68%)	*p*-value = 0.001148 (*p*-value < 0.05)
<Presentation date, *n* (%)				
Early presentation (≤7 days), *n* (%)	43 (58.1%)	36 (73.5%)	7 (28%)	
Late presentation (>7 days), *n* (%)	31 (41.9%)	13 (26.5%)	18 (72%)	*p*-value = 0.00046 (*p*-value < 0.05)
Bacterial growth, *n* (%)				
No bacterial growth, *n* (%)	25 (33.8%)	17 (34.7%)	8 (32%)	
Positive bacterial growth, *n* (%)	49 (66.2%)	32 (65.3%)	17 (68%)	*p*-value = 1.0 (*p*-value > 0.05)
Polymicrobial infected joints, *n* (%)	Total (n = 49)	Outcome—Favorable (n = 32)	Outcome—Unfavourable (n = 17)	
Single bacteria isolated, *n* (%)	36 (73.5%)	26 (81.3%)	10 (58.8%)	
Multiple bacteria isolated, *n* (%)	13 (36.5%)	6 (18.7%)	7 (41.2%)	*p*-value = 0.090546 (*p*-value > 0.05)
Drug resistant bacteria, *n* (%)	Total (n = 49)	Outcome—Favourable (n = 32)	Outcome—Unfavourable (n = 17)	
Non-MDR bacteria, *n* (%)	39 (79.6%)	27 (84.4%)	12 (70.6%)	
At least one MDR bacteria, *n* (%)	10 (20.4%)	5 (15.6%)	5 (29.4%)	*p*-value of 0.254371 (*p*-value > 0.05)

## Data Availability

The dataset is available on request from the authors.
